# Anthropogenic Versus Natural Controls on Metals in Surface Sediments from the Zhejiang Island Sea Area: Evidence from Historical Baseline and PMF Analysis

**DOI:** 10.3390/toxics14080669

**Published:** 2026-07-28

**Authors:** Ruirui Wang, Yanlong Gan, Jian Hu, Xinyu Mi, Jianyu Ni, Zhiyong Liu, Wenhan Cheng, Chuanyu Chang

**Affiliations:** 1 School of the Environment and Safety Engineering, Jiangsu University, Zhenjiang 212013, China; wangruirui@ujs.edu.cn (R.W.);; 2 Key Laboratory of Submarine Geosciences, Second Institute of Oceanography, Ministry of Natural Resources, Hangzhou 310012, China; 3 State Key Laboratory of Radiation Medicine and Protection, School of Radiation Medicine and Protection, Collaborative Innovation Center of Radiological Medicine of Jiangsu Higher Education Institutions, Biomedical Basic Research Center (BBRC) of Jiangsu, Soochow University, Suzhou 215123, China; 4 Anhui Provincial Key Laboratory of Hazardous Factors and Risk Control of Agri-Food Quality Safety, College of Resource & Environment, Anhui Agricultural University, Hefei 230036, China; 5 Laboratory of Karst Environmental Evolution and Ecological Security, Institute of Geochemistry, Chinese Academy of Sciences, Guiyang 550081, China

**Keywords:** historical baseline, metals, ecological risk assessment, surface sediments, source analysis, Zhejiang island sea area

## Abstract

Rapid industrialization, shipping, and petrochemical development have increased metal concentrations in coastal island ecosystems of eastern China. However, historical baseline information prior to large-scale coastal development remains scarce. This study investigated the concentrations and ecological risks of metals (Sr, Ni, V, Cr, Cu, Zn, Cd, Ba, Pb) in surface sediments collected in 2008 from the Zhejiang island sea areas, and used principal component analysis (PCA) and positive matrix factorization (PMF) to identify potential sources and estimate their contributions. Results showed that metals posed low to moderate ecological risks, with elevated risk levels occurring in Liuheng, Zhoushan, Daxie Island sea areas. Ni, Sr, Cu, Zn and Cd showed a certain degree of enrichment, whereas Cd presented the highest ecological risk. Three possible sources were identified: industrial activities (29.92%), combustion–lithological source (36.51%), and natural weathering (33.57%). Pb, Zn, Cu, and Cd appear to be primarily derived from industrial inputs, whereas V, Ni, and Cr showed a closer association with combustion–lithological sources. These results provided a historical geochemical baseline for this rapidly industrializing island sea areas in China and a reference framework for evaluating long-term environmental changes associated with coastal development, port expansion, and maritime activities in the East China Sea.

## 1. Introduction

Metals, characterized by persistence, high toxicity, and non-degradability, may pose serious risks to ecosystems and human health if their environmental concentrations exceed ecological thresholds [1,2,3]. In coastal ecosystems, the ecological risk of metals arises not only from their inherent toxicity, but also from the fact that the concentrations introduced by anthropogenic activities often exceed natural background levels [3,4]. Upon entering aquatic environment, metals predominantly accumulate in sediments via processes such as adsorption and flocculation [5,6]. Alterations in the aquatic environment can lead to the re-release of metals from sediments into overlying waters, resulting in secondary pollution that adversely affects aquatic organisms and human health via the food chain [7,8].

Islands, located in the transitional zone of land–sea interaction, are highly sensitive to both natural environmental changes and anthropogenic disturbances. Coastal regions, owing to their favorable natural conditions, have evolved into densely populated centers of economic activity and also serve as important sinks for anthropogenic pollutants, including metals [9,10]. Moreover, the rapid economic development of islands, especially the emergence of port-related sectors such as shipbuilding, green petrochemicals, and energy storage and transfer, as well as the growth of tourism and aquaculture, has contributed to the introduction of metals from coastline into surrounding sea areas, significantly intensifying the accumulation of metals in sediments from the island sea areas [11,12]. Alterations in the sedimentary environment may result in the re-release of metals from sediments into the water, leading to secondary contamination [3,7]. Therefore, systematic analysis of metal concentrations, spatial distribution, sources, and ecological risks in sediments from island sea areas not only serves as an important basis for elucidating the impact of regional human activities on the environment, but also provides a scientific foundation for developing effective marine environmental management strategies to safeguard the ecological health of the islands.

The islands of Zhejiang Province are located in the East China Sea, including Zhoushan Island, Aoshan Island, Putuoshan Island, Zhujiajian Island, Dongji Island, Nanji Island, and Huabiao Island, etc. This sea area receives freshwater inputs from multiple inland rivers, including the Yangtze River, Qiantang River, Ou River, and Min River, which transport abundant terrestrial nutrients and serve as a vital food source for marine organisms, establishing this area as one of China’s major fishing grounds [13,14]. Recent studies on the distribution characteristics and pollution risks of metals in offshore sediments have primarily focused on areas significantly affected by terrestrial inputs, such as bays and estuaries [4,15]. In contrast, islands, as distinct geographical units influenced by both direct anthropogenic activity and open seawater exchange, remain poorly understood with respect to the distribution patterns, accumulation characteristics, and potential ecological risks of metals in their sediments. Therefore, this study aimed to investigate the concentrations, spatial distribution patterns, ecological risks, and potential sources of metals in surface sediments from the Zhejiang island sea areas. More importantly, because the samples were collected before the rapid expansion of petrochemical industries, large-scale port construction, and intensive shipbuilding activities that occurred after 2010, this dataset provides a valuable historical baseline for evaluating long-term anthropogenic impacts on the island marine environment. Establishing such a baseline is essential for distinguishing natural geochemical variability from subsequent industrial contamination and for supporting future environmental assessments and management strategies in the Zhejiang island sea areas.

## 2. Materials and Methods

### 2.1. Sampling Collection

**Figure 1:**
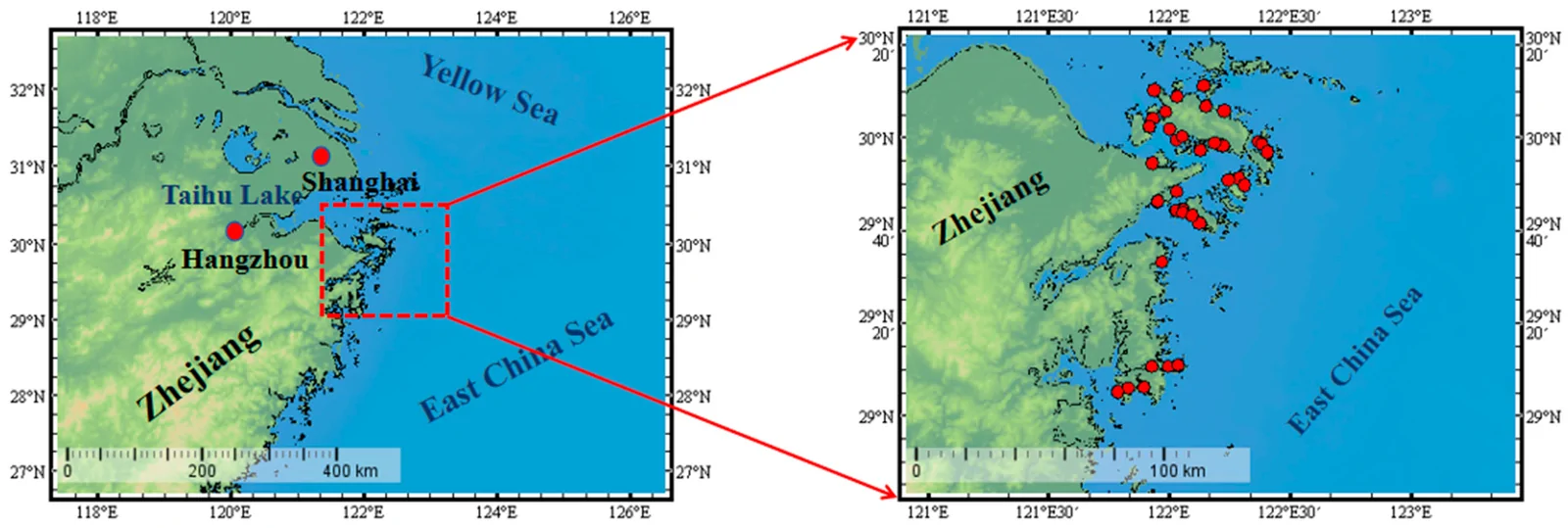
Sampling sites of surface sediments from the Zhejiang island sea areas.

The surface sediment samples from the Zhejiang island sea areas were collected within the coordinate range of 121°47.10′–122°24.48′ E and 29°05.20′–30°11.30′ N (October–December 2008) (Figure 1). Eighty-two surface sediment samples were collected using a grab-type sediment sampler at water depths ranging from approximately 10 to 40 m. At each sampling site, approximately 250 g of undisturbed sediment was collected from the 0–5 cm depth using a plastic sample spoon, placed into polyethylene sample bags and stored in a 4 °C refrigerator. Stones, shells, and other impurities were removed from the samples, which were then dried at 50 °C. The dried samples were ground to 200 mesh for subsequent chemical analysis.

### 2.2. Analysis Method

Carefully weigh 50 mg of sediment sample and soil reference standards (GSS–7 and GSS–13) into clean 7 mL Teflon^®^ PFA screw-cap vials; then, add 3 mL mixed acid (1:2 mixture of high-purity H_2_O_2_ and concentrated HNO_3_) to remove organic matter, and carefully evaporate the mixture to dryness on a low-temperature hotplate. Repeat this process 2–3 times to ensure complete removal of organic matter. Subsequently, use 4 mL mixed acid (1:3 concentrated HNO_3_ and HF) to digest samples at 140 °C for approximately 48 h, until a significant quantity of white flocculent matter is observed in the solution. To ensure complete digestion of the samples, treat them with ultrasound for 1 h, and then continue heating for 12 h. Repeat the ultrasonication and heating 2–3 times until all samples are completely dissolved. Then, evaporate the samples to complete dryness on a hot plate at 140 °C. Add 4 mL of aqua regia to the evaporated samples and heat at 100 °C for 12 h. After evaporating the samples to dryness on a hotplate at 120 °C, repeatedly treat the solution with 1:2 mixture of high-purity H_2_O_2_ and concentrated HNO_3_ until the solutions are clear.

Metal concentrations were analyzed at the University of Science and Technology of China using an inductively coupled plasma mass spectrometer (ICP–MS, iCAP RQ, Thermo Scientific, Bremen, Germany, high sensitivity and low detection limits). To ensure analytical precision, geochemical standard reference soils (GSS–7 and GSS–13) were analyzed, achieving a relative standard deviation within 5–10% and recoveries ranging from 80% to 110%. In addition, after every 20 samples, standard solutions (10 μg/L, 50 μg/L, 100 μg/L, 500 μg/L) were measured to correct for instrument drift, with each measurement performed three times. The detection limits were 0.05 μg/L for Cu and V, 0.003 μg/L for Pb and Ni, 0.005 μg/L for Cd, Cr and Sr, 0.1 μg/L for Zn, 0.01 μg/L for Ba. Both field blanks and measurement blanks were below the detection limits.

### 2.3. Contamination and Risk Assessment Methods

The geological accumulation index (*I*_geo_) [16] is often used to assess the degree of metal pollution in the environment:

$$I_{geo}={log}_{2}\left(\frac{C_{M}}{1.5\times C_{B}}\right)$$

C_M_ represents the metal element concentrations of the samples, and C_B_ represents the background values of the metal elements (the background values of sediments in the offshore area of Zhejiang are detailed in Table 1). Based on *I*_geo_ values, pollution levels are divided into seven levels from 0 to 6, ranging from 0 (*I*_geo_ < 0, unpolluted) to 6 (*I*_geo_ > 5, extremely polluted).

The concentration of metals in sediments does not reflect the degree of metal enrichment [17]. Enrichment factors (EFs) are used to evaluate the pollution level of trace metal elements by comparing them with background levels [18]. In addition, geochemical normalization using conserved elements such as Al, Li, Ti, Fe, V, and Mn can eliminate the effects of grain size and minerals on trace metal concentrations [19].

$$EF=\frac{{\left(\frac{M}{N}\right)}_{sample}}{{\left(\frac{M}{N}\right)}_{background}}$$

M represents the metal concentrations of the samples, and N represents the normalized element concentrations. In this study, Ti was used as the geochemical normalized element for calculating the EF value (the background value of Ti is 4889.00 mg/kg) [20]. EF value less than 1 indicates that weathered crustal materials are likely the primary source of metals; EF value greater than 1 indicates that a large portion of metals are from anthropogenic sources. The EF categories are defined as follows: EF ≤ 1, no enrichment; 1 < EF ≤ 2, slight enrichment; 2 < EF ≤ 5, moderate enrichment; 5 < EF ≤ 20, high enrichment; and 20 < EF ≤ 40, extremely high enrichment [21].

The potential ecological risk index (PERI) is commonly used to measure the potential harm of metals to ecosystems [22]. This method considers factors such as the pollution degree of metals, toxicity, and environmental impact, and can quantitatively evaluate the potential ecological risk parameter ($E_{r}^{i}$) for a single metal and the overall potential ecological risk index (*RI*) for multiple metals.

$$RI=\sum_{i=1}^{n}E_{r}^{i}=\sum_{i=1}^{n}T_{r}^{i}\times C_{f}^{i}/C_{n}^{i}$$

where $C_{f}^{i}$ and $C_{n}^{i}$ are the measured value and background values of metal i, respectively; $E_{r}^{i}$ is the potential ecological risk index for metal i; and $T_{r}^{i}$ is the toxicity coefficient of metal i, where the toxicity coefficients of Cr, Cd, Ni, Pb, Cu and Zn are 2, 30, 5, 5, 5 and 1, respectively [22,23,24]. $E_{r}^{i}$ is divided into five levels: light ($E_{r}^{i}$ < 40), moderate (40 ≤ $E_{r}^{i}$ < 80), fairly strong (80 ≤${\;E}_{r}^{i}\;$< 160), strong (160 ≤$E_{r}^{i}\;$< 320), and extreme ($E_{r}^{i}\;$≥ 320). *RI* is divided into four levels: light (*RI* < 150), moderate (150 ≤ *RI* < 300), fairly strong (300 ≤ *RI* < 600), and strong (*RI* ≥ 600).

### 2.4. Statistical Analysis

The statistical analysis of the data in this study was performed using IBM SPSS 26.0 software and was plotted by the Origin 2025 software, including the calculation of maximum, minimum values, standard deviation, and coefficient of variance for each parameter. A one-sample *t*-test was used to examine the sample independence between the metal concentrations and background values (*p* < 0.05).

The source apportionment of metals in surface sediments was conducted by the receptor models PMF (EPA PMF Version 5.0). All metal concentrations in this study were above the detection limits. The uncertainty data was calculated as $uncertainty=\sqrt{{\left(error\;fraction\times concentration\right)}^{2}+{\left(detection\;limit\right)}^{2}}$, with the error fraction set between 0.1 and 0.2 based on analytical precision. To evaluate the rotational ambiguity and determine the optimal number of factors, we conducted displacement, bootstrap, and bootstrap-enhanced displacement analyses. To determine the optimal number of source factors, the model was run with the number of factors set from 3 to 5, each with 20 iterations. The result with 3 factors was ultimately selected, as it yielded a Qtrue/Qexp value of 0.63, which fell within the ideal range of 0.5–1.5. The residuals of all elements fell within [−3, 3] and followed a normal distribution, indicating the best fitting performance and stable calculation results. The robustness of the 3 factors was further evaluated through a series of diagnostic analyses. Rotational ambiguity was assessed using the Fpeak parameter by exploring rotations from −1.0 to +1.0 in increments of 0.5. The base model (Fpeak = 0) provided the most interpretable factor profiles with minimal Q-value changes (< 5%), indicating rotational stability. Additionally, 100 bootstrap runs were performed with a minimum correlation R-value of 0.6, yielding a bootstrap mapping rate exceeding 80%, which further confirms the stability and reliability of the model.

Analysis using this model showed that the R^2^ values for Ni, V, Cr, Cu, Zn, Ba, Pb, Cd, and Sr were 0.85, 0.83, 0.90, 0.94, 0.91, 0.93, 0.90, 0.38, and 0.27, respectively, with an overall R^2^ of 0.89, indicating a good fit. The relatively poor fit for Cd (R^2^ = 0.38) was mainly due to its relatively high intraspecies relative variability (CV = 28.61%), higher than that of other metals except Pb (Table 1). In addition, approximately seven-fold dynamic range of Cd concentrations (0.10–0.67 mg/kg) resulted in a structurally unfavorable low signal-to-noise ratio, leading to a small weight assigned to Cd in the PMF objective function, which made it difficult for the model to effectively capture the variation characteristics of Cd [25]. The low fit for Sr (R^2^ = 0.27) was primarily attributed to its low coefficient of variation (14.8%) (Table 1) and uniform spatial distribution, as well as the fact that Sr and Ba, both alkaline earth metals, share similar geochemical behavior. When allocating high concentrations of Ba, the model absorbed part of the variation in Sr, thereby reducing the independent fitting performance for Sr. The contributions of Cd and Sr to the three factors were extremely low (Cd < 0.05%, Sr mainly occurred in natural sources) (Table S1), which did not affect the overall conclusions regarding source identification and factor interpretation.

It should be noted that PMF, as a statistical model based on concentrations, is subject to factors such as data quality, factor numbers, and rotational ambiguity. Therefore, its source apportionment results should be understood as the best inference based on the available data, rather than as an exact quantification of true source contributions.

**Table 1:** Statistical description of metal concentrations in surface sediments from the Zhejiang island sea areas (82 samples; mg/kg).

Metals	Sr	Ni	V	Cr	Cu	Zn	Cd	Ba	Pb
Min	118.60	16.10	70.89	44.31	14.29	57.98	0.10	349.20	17.47
Max	267.45	70.26	141.02	139.47	82.48	242.40	0.67	1076.45	111.89
Average	152.31	35.70	110.94	88.81	29.01	97.08	0.23	426.30	26.30
Standard deviation (S.D.)	22.51	7.82	15.96	15.62	8.24	24.93	0.06	88.96	11.86
Variable coefficient (C.V.)	14.78	21.89	14.39	17.59	28.42	25.68	28.61	20.87	45.10
Background value	120.00 *	20.00 **	108.00 *	92.30 *	28.40 *	92.40 *	0.11 *	476.00 *	26.40 *

* The background values of sediments are from [20]; ** the background values of sediments are from [26].

## 3. Results and Discussion

### 3.1. Concentrations and Distribution Patterns of Metals in Surface Sediments

**Figure 2:**
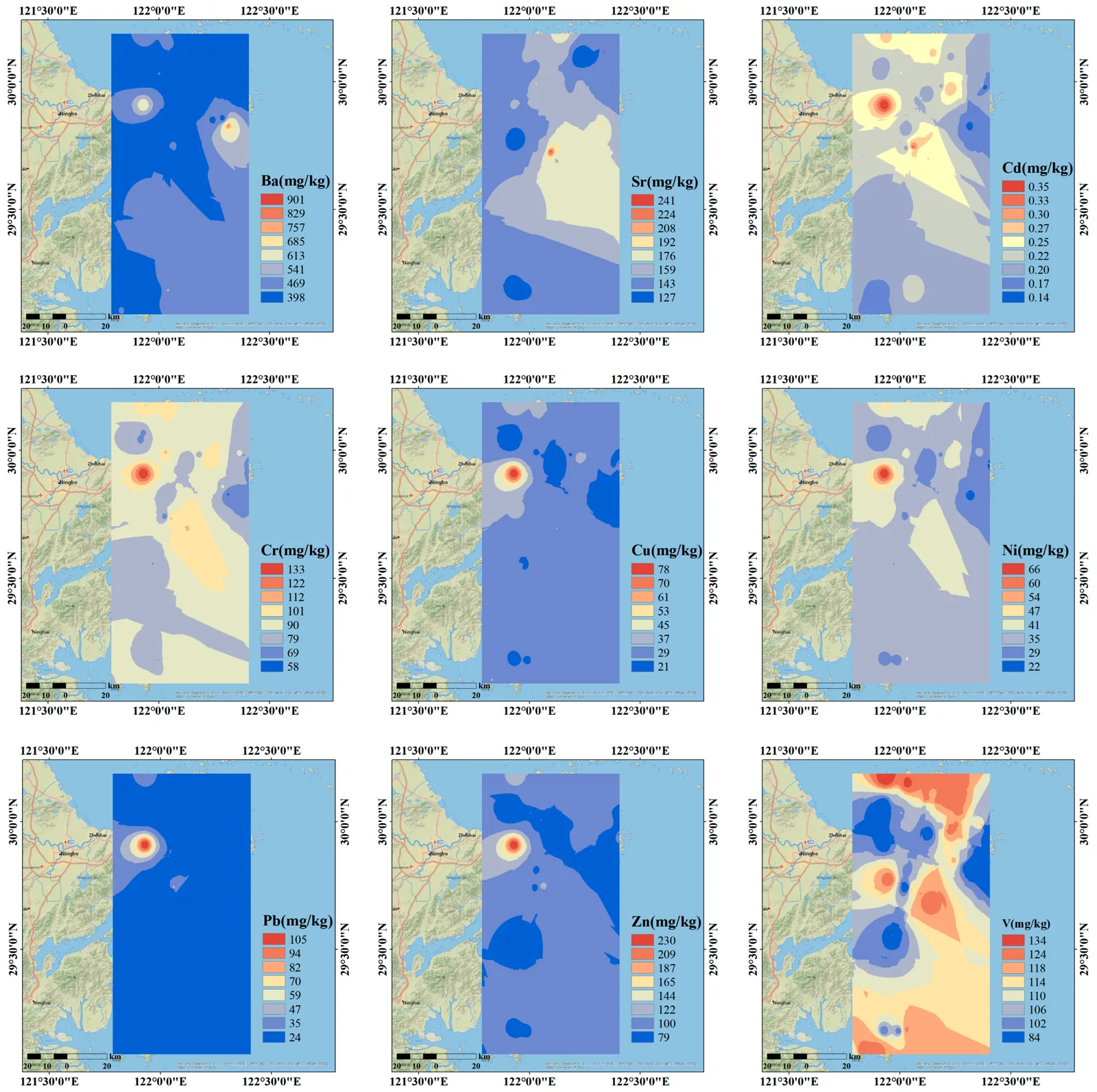
Spatial distribution of metals (Ba, Sr, Cd, Cr, Cu, Ni, Pb, Zn, V) in surface sediments from the Zhejiang island sea areas.

The metal concentrations in surface sediments from different island sea areas of Zhejiang were shown in Table 1 and Table S2 and Figure 2. The results showed that metal concentrations varied among these areas.

The Ni concentrations ranged from 16.10 to 70.26 mg/kg. Relatively low Ni concentrations (16.10−34.94 mg/kg) were found in the Taohua Island sea area, Zhoushan City; while the highest Ni concentration (70.26 mg/kg) was found in the Daxie Island sea area, Ningbo City. Among the studied islands, the Ni concentrations in surface sediments from the Zhoushan and Liuheng Island sea areas in Zhoushan City were relatively higher than those from other island sea areas, ranging from 30.42 to 46.63 mg/kg and 29.69 to 46.69 mg/kg, respectively. The Ni concentrations in surface sediments from the Cezi (27.21 mg/kg), Jintang (26.22–29.84 mg/kg), and Aoshan (25.46−28.56 mg/kg) Island sea areas were relatively low.

The Cr concentrations ranged from 44.31 to 139.47 mg/kg, with the highest value (139.47 mg/kg) in the Daxie Island sea area, Ningbo City, and the lowest value (44.31 mg/kg) in the Taohua Island sea area, Zhoushan City. Furthermore, the Cr concentrations in surface sediments from the Xiushan, Zhoushan, and Liuheng Island sea areas in Zhoushan City were relatively higher than those from other island sea areas, ranging from 89.43 to 100.83 mg/kg, 76.21 to 114.82 mg/kg, and 81.29 to 121.14 mg/kg, respectively. In contrast, the Cr concentrations in surface sediments from the Jintang (70.45–79.89 mg/kg) and Zhujianjian (59.27–89.63 mg/kg) Island sea areas were relatively low.

The Sr concentrations in surface sediments were relatively uniform, ranging from 118.60 to 267.45 mg/kg. The highest value was found in the Liuheng Island sea area (267.45 mg/kg), and the lowest value was found in the Zhoushan Island sea area (118.60 mg/kg).

The V concentrations ranged from 70.89 to 141.02 mg/kg, with the highest value found in the Zhoushan Island sea area (141.02 mg/kg), and the lowest value found in the Taohua Island sea area (70.89 mg/kg). Furthermore, the V concentrations in surface sediments from the Xiushan, Zhoushan, Liuheng Island sea areas in Zhoushan City, and the Tantoushan Island sea area in Ningbo City were relatively higher than those from other island sea areas, ranging from 116.83 to 132.35 mg/kg, 87.66 to 141.02 mg/kg, 96.07 to 131.19 mg/kg, and 112.51 to 126.38 mg/kg, respectively. In comparison, the V concentrations in surface sediments from the Cezi (91.23 mg/kg), Daxie (94.19 mg/kg), and Fodu (73.66–99.04 mg/kg) Island sea areas were relatively low.

The Cu concentrations ranged from 14.29 to 82.48 mg/kg, with the highest value found in the Daxie Island sea area, Ningbo City (82.48 mg/kg), and the lowest value found in the Taohua Island sea area, Zhoushan City (14.29 mg/kg). Furthermore, the Cu concentrations in surface sediments from the Zhoushan and Meishan Island sea areas of Ningbo City were relatively high, ranging from 23.33 to 41.60 mg/kg and 25.75 to 38.07 mg/kg, respectively. In contrast, the Cu concentrations in surface sediments from the Jintang (18.50–23.16 mg/kg) and Aoshan (17.46–22.53 mg/kg) Island sea areas were relatively low.

The Zn concentrations ranged from 57.98 to 242.40 mg/kg, with the highest value found in the Daxie Island sea area, Ningbo City (242.40 mg/kg) and the lowest value found in the Putuo Island sea area, Zhoushan City (57.98 mg/kg). The Zn concentrations in surface sediments from the Zhoushan and Tantoushan Island sea areas were relatively higher than those from other island sea areas, ranging from 79.72 to 137.12 mg/kg and 103.72 to 108.33 mg/kg, respectively. In contrast, lower concentrations were observed in the Jintang (68.06–91.31 mg/kg) and Aoshan (66.26–80.85 mg/kg) Island sea areas.

The Cd concentrations ranged from 0.10 to 0.67 mg/kg, with the highest value found in the Liuheng Island sea area (0.67 mg/kg) and the lowest value found in the Putuo Island sea area (0.10 mg/kg). Furthermore, the Cd concentrations in surface sediments from the Xiushan, Zhoushan, and Liuheng Island sea areas were relatively high, ranging from 0.25 to 0.27 mg/kg, 0.20 to 0.30 mg/kg, and 0.18 to 0.67 mg/kg, respectively. In contrast, the Cd concentrations in surface sediments from other island sea areas were relatively consistent.

The Ba concentrations in surface sediments were relatively uniform, ranging from 349.20 to 1076.45 mg/kg. The highest value was found in the Taohua Island sea area (1076.45 mg/kg), and the lowest value was found in the Liuheng Island sea area (349.20 mg/kg).

The Pb concentrations in surface sediments were relatively uniform, ranging from 17.47 to 111.89 mg/kg. The highest value was found in Daxie Island sea area, Ningbo City (111.89 mg/kg), and the lowest value was found in Jintang Island sea area, Zhoushan City (17.47 mg/kg).

Based on the above, the concentrations of Ni, Cr, Cu, Zn, and Pb in surface sediments from the Daxie Island sea area were significantly higher than those of other island sea areas, while the concentrations of Ni, Cr, V, and Cu in surface sediments from the Taohua Island sea area were relatively low (Table S2). Furthermore, the concentrations of Sr (118.60–267.45 mg/kg), Ni (16.10–70.26 mg/kg), and Cd (0.10–0.67 mg/kg) almost exceed the background values of the offshore area of Zhejiang (120.00 mg/kg, 20.00 mg/kg, and 0.11 mg/kg, respectively) (Table 1) [20,26], indicating the accumulation of these three elements in surface sediments. In contrast, the average concentrations of V, Cr, Cu, Zn, and Pb in surface sediments from some island sea areas were comparable to the background. While the average concentration of Ba was lower than the background value (Table 1). The variable coefficients could further reveal the degree of spatial heterogeneity of each element [4]. The variable coefficients for the nine metals ranged from 14.39% to 45.10%, with V having the lowest coefficient (14.39%) and Pb having the highest (45.10%) (Table 1), indicating that the spatial heterogeneity of Pb distribution was more prominent, while the spatial distribution of V showed more pronounced homogeneity.

Table 2 summarized the metal concentrations in surface sediments from some coastal areas of China, as well as the national Class I and Class II standards for marine sediment environmental quality in China. Compared with other coastal areas, the average concentrations of Ni, Cr, Cu, and Zn in surface sediments from the Zhejiang island sea areas were relatively high. The average Cd concentration in surface sediments from the Zhejiang island sea areas was significantly higher than those in the coastal areas of southern Zhejiang, Hebei, the Shandong Peninsula, and Fujian [12,15,27,28], but significantly lower than those in the coastal areas of Lianyungang and the Bohai Sea [4,29]. The average Pb concentration in surface sediments from the Zhejiang island sea areas was significantly higher than those in the coastal areas of Lianyungang, the Shandong Peninsula, the Yellow Sea, and the Bohai Sea [4,27,29], but significantly lower than those in the coastal areas of Fujian [28].

Compared with the Class I standard for marine sediment quality in China, there were significant differences in the compliance status of different metals in surface sediments from the Zhejiang island sea areas [30]. The average concentrations of Cu, Zn, Cd, and Pb were all below the Class I standard limit, while the average concentration of Cr was relatively high. The average concentrations of all metals were below the Class II standard reference values. At the sampling sites, the average concentrations of Cr, Cu, Zn, Cd, and Pb were below the Class II standard. Regarding compliance with the Class I standard, the compliance rates for Cu, Zn, Cd, and Pb were relatively high, with the vast majority of sampling points meeting the Class I quality standard (Table 2). However, for Cr, although all sites met the Class II quality standard, only 26.83% of the sites reached the Class I standard, indicating that a certain degree of Cr pollution had occurred in some areas of the sediments of Zhejiang islands.

According to the spatial distribution (Figure 2), Cd, Cr, Cu, Ni, Pb, and Zn exhibited similar spatial distribution characteristics, with their concentrations in surface sediments from the Daxie Island sea area in Ningbo City being significantly higher than those from other island sea areas, indicating that the spatial distribution of these metals might be primarily influenced by local anthropogenic activities on the islands. In contrast, the spatial distribution of V showed patchy patterns, reflecting source characteristics of diversity and spatial continuity.

### 3.2. Assessment of Metal Pollution

To determine the enrichment levels of metals in surface sediments from the Zhejiang island sea areas, this study calculated the enrichment factors (EF values, Table S3) of nine elements. The results showed that the average EF values of these nine metals, from highest to lowest, were as follows: Cd (1.93) > Ni (1.67) > Sr (1.20) > Zn (0.99) > V (0.96) = Cu (0.96) > Pb (0.94) > Cr (0.90) > Ba (0.85). The average EF values of Zn, V, Pb, Cr, Cu and Ba were all less than 1, indicating that these elements did not show significant enrichment on an overall average basis. However, based on the EF values of individual sampling points, the percentages of samples with EF > 1 for the above elements were Zn (40.24%), V (35.37%), Pb (23.17%), Cr (19.51%), and Ba (9.76%), respectively. This indicated that, although the overall enrichment levels of these metals were relatively low, slight enrichment trends were still observed at some sampling sites. The spatial variability may be attributed to lithological differences in the study area [31], but the influence of local anthropogenic inputs cannot be entirely excluded. Therefore, the interpretation of these results should be conducted with caution, taking into account the regional geological background. Furthermore, the average EF values of Cd, Ni and Sr were significantly higher than those of the other elements, indicating a certain degree of anthropogenic influence. Specifically, the average EF value of Cd was 1.93, with a maximum value of 5.19, and the EF values of all samples were greater than 1, indicating that Cd had reached a slight to high enrichment level, and that anthropogenic activities might have significantly influenced their enrichment. Ni and Sr also showed similar enrichment trends, with average EF values of 1.67 and 1.20, respectively, and maximum values of 3.66 and 2.18, respectively. Notably, samples with EF values greater than 1 for Ni and Sr accounted for as much as 97.56% and 98.78%, respectively, indicating that these metals also exhibited slight to moderate enrichment in surface sediments from the Zhejiang island sea areas, and that anthropogenic input might have significantly influenced their enrichment.

Based on the calculation results of the geological accumulation index (*I*_geo_), the average *I*_geo_ values of Ni and Cd were greater than 0, indicating that these two elements generally exhibited slight enrichment and suggested mild contamination, although the influence of regional geology cannot be excluded. Moreover, among the samples with *I*_geo_ values greater than 0, Ni accounted for 79.27% and Cd accounts for 95.12%, further indicating that Ni and Cd showed potential enrichment and possible mild contamination. The average EF value of Sr was greater than 1 (Table S3), while its average *I*_geo_ value was less than 0, indicating that Sr showed enrichment characteristic but did not reach the level of pollution. For Sr, Cr, Cu, Zn, Ba, and Pb, although their *I*_geo_ values were all less than 0, indicating no overall pollution; however, the *I*_geo_ values of some samples of these elements were greater than 0 (Table S4), suggesting slight pollution in certain surface sediments of Zhejiang island sea areas.

To further assess the ecological risk of metals in surface sediments from the Zhejiang island sea areas, this study calculated the ecological risk factor ($E_{r}^{i}$) and risk index (*RI*) for six metals (Table S5). The average $E_{r}^{i}$ values, from high to low, were as follows: Cd > Ni > Cu > Pb > Cr > Zn. Except for Cd, the average $E_{r}^{i}$ values of the other five metals were all less than 40, indicating that their ecological risks were relatively low based on the concentration calculations. The average $E_{r}^{i}$ value of Cd was 61.77, ranging from 26.47 to 183.12, making it the element with the highest ecological risk in the study area. Among the sampling sites, 42 sites had Cd risk at a moderate ecological risk level (40 < $E_{r}^{i}$ < 80); 3 sites had Cd risk at a high ecological risk level (80 < $E_{r}^{i}$ < 160), located in the Zhoushan and Daxie Island sea areas; and 1 site had Cd risk at a very high ecological risk level (160 < $E_{r}^{i}$ < 320), located in the Liuheng Island sea area in Zhoushan City. These results indicated that the ecological risk of Cd in surface sediments was relatively prominent and required attention. However, it should be noted that the *I*_geo_ and $E_{r}^{i}$ values in this study were calculated using sediment concentrations without grain-size or clay-content normalization. To evaluate the potential influence of lithogenic factors, the *I*_geo_ values of Cd and Ni were regressed against Ti (a conservative element). The regression yielded R^2^ values of 0.18 for Cd and 0.30 for Ni (both *p* < 0.05), indicating that lithogenic control contributes to the enrichment of these metals, but is not the sole controlling factor, and that other sources (e.g., anthropogenic inputs) may also play an important role (Figure S1). Therefore, due to the lack of grain-size normalization, the *I*_geo_ and $E_{r}^{i}$ values should be interpreted as indicative measures of potential contamination rather than as definitive conclusions.

**Figure 3:**
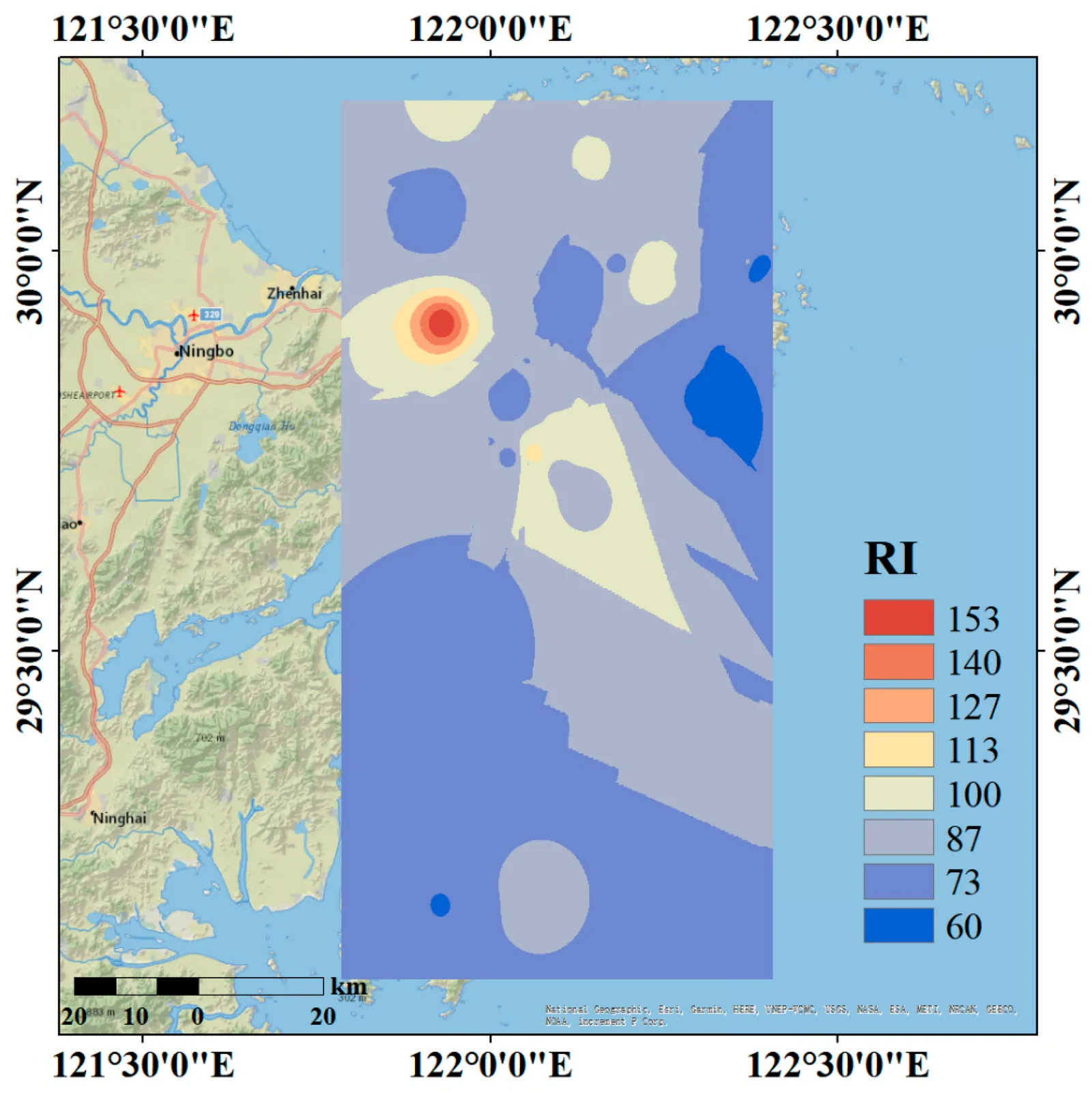
*RI* diagram of six metals (Ni, Cr, Cu, Zn, Cd, Pb) in surface sediments from the Zhejiang island sea areas.

Based on the ecological risk index (*RI*) calculation results for six metals (Table S5), the *RI* values of all sites were below 300, indicating that the overall ecological risk of metal pollution was generally at a low to moderate level. The spatial distribution of *RI* was highly consistent with the distribution of the main risk element Cd (Figure 2 and Figure 3), and the high-risk areas were mainly located in the Liuheng, Zhoushan, and Daxie Island sea areas, further confirming that Cd might be a key contributor to the potential regional ecological risk in the studied areas. Given the potential biotoxicity of Cd and its significant enrichment in sediments, further investigation (including grain-size normalized assessment) is warranted to confirm whether the Cd concentrations pose actual ecological risks. Since Cd can be transmitted through the food chain, posing a threat to marine ecosystems and human health [32], if such risks are confirmed, enhanced long-term monitoring and targeted management measures are recommended for the high-Cd areas, including the Liuheng, Zhoushan, and Daxie Island sea areas.

### 3.3. Source Identification of Metals

#### 3.3.1. Correlation Analysis

**Figure 4:**
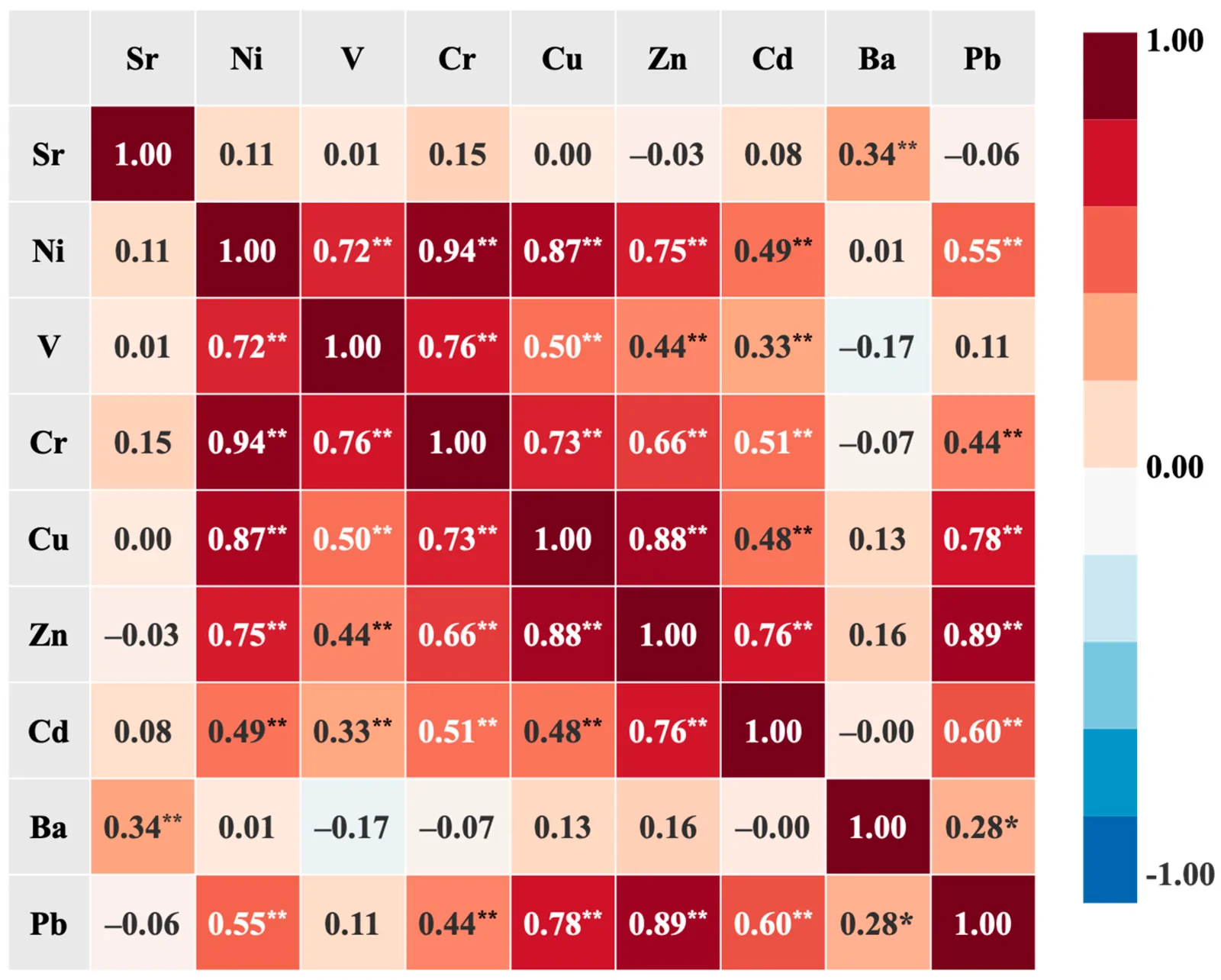
Correlation matrix heatmap of metals in surface sediments from the Zhejiang island sea areas (** indicates that the correlation is significant at the *p* = 0.01 level (two-tailed); * indicates that the correlation is significant at the *p* = 0.05 level (two-tailed)).

Pearson correlation analysis helps to reveal the degree of closeness of relationships between variables [33,34]. Figure 4 lists the correlation coefficients of nine metals in surface sediments from the Zhejiang island sea areas. Among them, Ni, V, Cr, Zn, and Cu showed strong positive correlations. Cu and Pb, as well as Zn and Cd, also showed strong positive correlations. Their similar spatial distribution characteristics further support that they had a common source or similar enrichment mechanisms (Figure 2). Additionally, Pb showed a certain positive correlation with Ni, Cr, and Ba, with correlation coefficients ranging from 0.28 to 0.55. Pb showed weak positive correlation with V (r = 0.11) and negative correlation with Sr (r = −0.06). Ba showed negative correlation with V, Cr, and Cd, indicating that Ba might have different sources from these elements (Figure 4). Notably, Pb had a high coefficient of variation (45.1%) (Table 1), and the strength of its correlation with different elements varied greatly, suggesting that there might be multiple sources of Pb in surface sediments.

#### 3.3.2. Coupled Analysis of PCA and PMF

**Figure 5:**
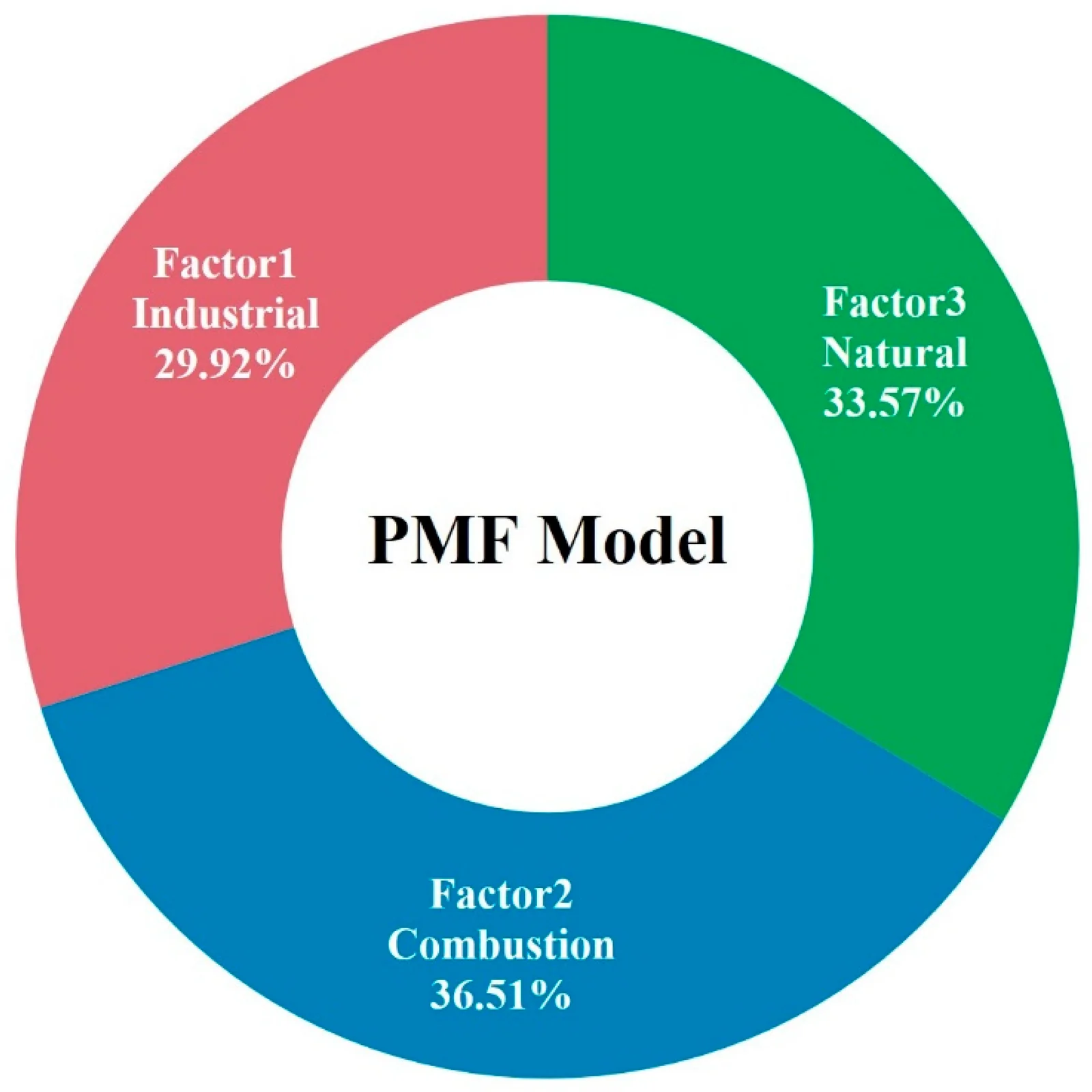
Contribution rates of different sources to metals in surface sediments from Zhejiang island sea areas.

Principal component analysis (PCA) and positive matrix factorization (PMF, *p* = 3) were applied to nine metals in surface sediments from the Zhejiang island sea areas. PCA results showed that all three principal components had eigenvalues greater than 1, with a cumulative explained variance of 84.17%, indicating that the metals were primarily derived from three sources (Table S6). The three factors resolved by PMF corresponded exactly to the three principal components of PCA in source identification. Moreover, PMF can directly output the percentage contribution of each element to each factor, compensating for the limitation that PCA only provides loading coefficients. It should be noted that source apportionment itself is inherently complex, and the results of PCA and PMF models should be considered as reference evidence for source identification rather than as definitive conclusions. Based on the combined analysis of these two models, the PMF analysis results indicated that the nine metals in surface sediments from the Zhejiang island sea area might be grouped into three sources: an industrial source centered on Pb–Zn–Cu–Cd (29.92%), a combustion–lithological source characterized by V–Ni–Cr (36.51%), and a natural weathering source dominated by Sr–Ba (33.57%) (Figure 5).

**Figure 6:**
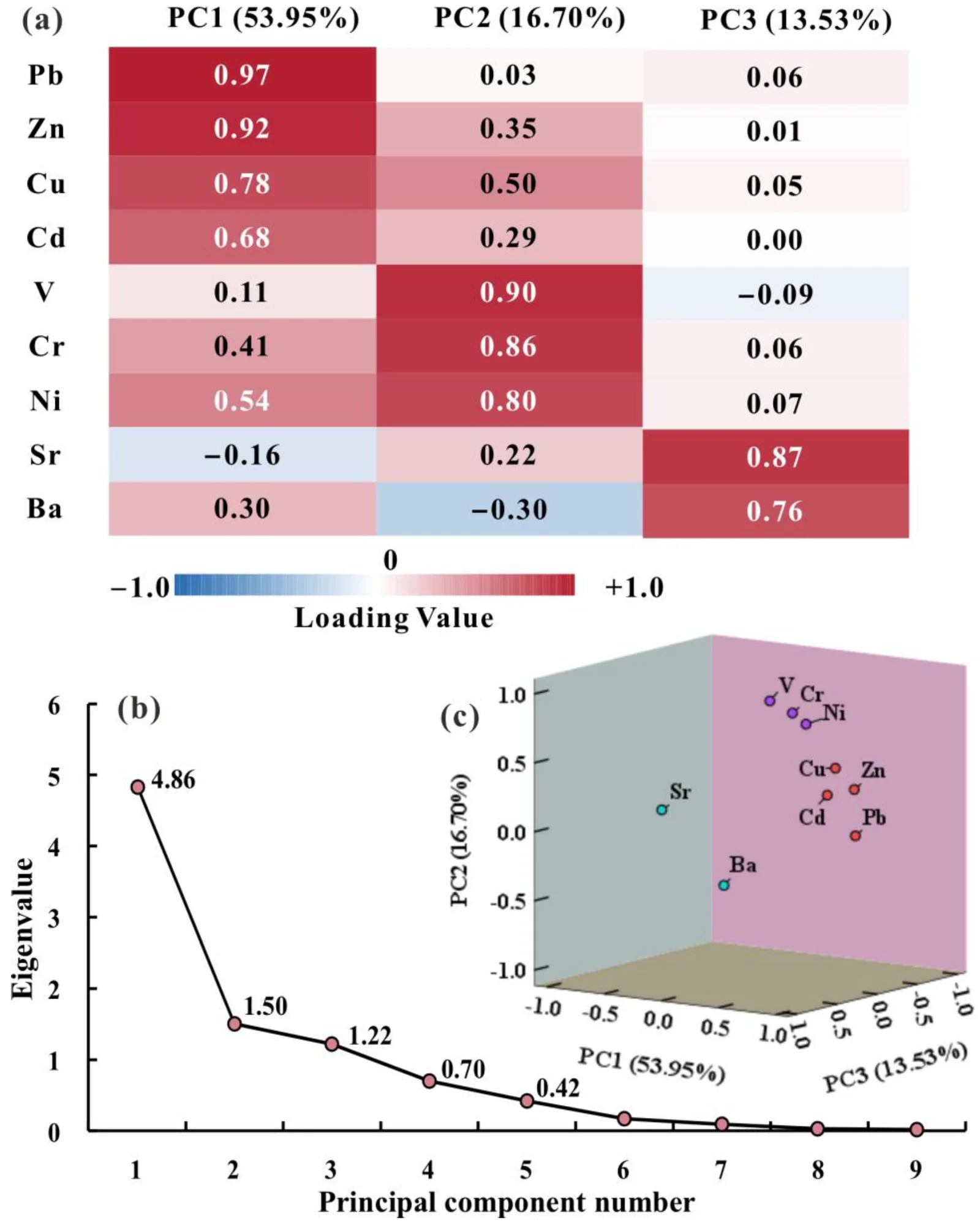
Graphical representation of the PCA (**a**), scree plot (**b**), and variance contribution (**c**) of metals in surface sediments from the Zhejiang island sea areas.

**Figure 7:**
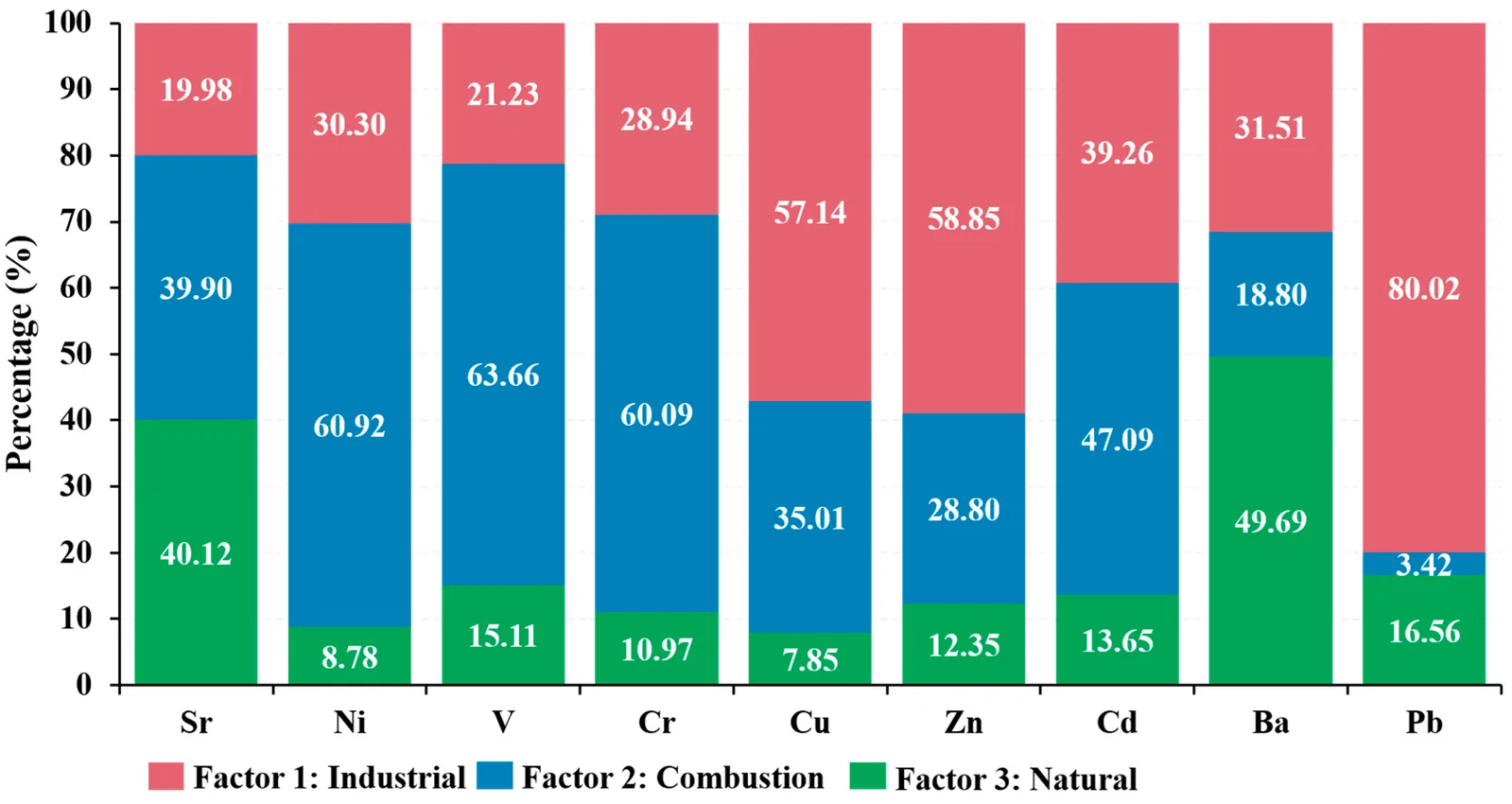
The proportions of the three factors in each metal (%) in surface sediments from the Zhejiang island sea areas.

The variance contribution rate of PC1 was 53.95%, with high loadings of Pb (0.97), Zn (0.92), Cu (0.78), and Cd (0.68) (Table S6, Figure 6). Compared with the background values of coastal sediments of Zhejiang, the average concentration of Cd was relatively higher, while those of Cu and Zn were generally consistent with the background values (Table 1). Furthermore, these elements also showed good consistency in their spatial distributions (Figure 2). Relevant studies have showed that Cu, Zn, and Pb mainly originate from iron and steel smelting, galvanized copper plating, anti-corrosion materials, and battery manufacturing, while Cd is typically associated with anti-corrosion materials and smelting [35,36,37]. In PMF, Factor 1 (F1) was characterized by dominant contributions from Pb (80.03%), Zn (58.85%), and Cu (57.14%), all exceeding 57%, and was therefore identified as an industrial source (Figure 7). This source likely reflects industrial activities occurring along the Zhejiang coast and islands. The strong association among Pb, Zn, Cu, and Cd suggests contributions from metal-processing industries, ship maintenance activities, anti-corrosion materials, electroplating operations, and related industrial emissions. However, because PMF is a receptor model based solely on concentration covariance, the identified source should be interpreted as a generalized industrial source rather than being attributed exclusively to a specific industrial sector. Among them, the contribution of Pb was as high as 80.03%, which was the highest industrial source contribution among all element-factor combinations, and may be related to the dense shipbuilding industry in the Zhoushan Archipelago. The extensive use of lead-based antirust paints (e.g., red lead paint, containing 60–90% Pb) in ship maintenance generates lead-containing dust during operations such as abrasive blasting and old paint removal, which then enters the marine environment through atmospheric deposition [35,38]. Pb showed an overwhelmingly dominant contribution only to F1 in the PMF analysis, indicating a highly specific input pathway, making it an ideal tracer for identifying shipbuilding industrial pollution. Zn contributed 58.85% to F1 and 28.80% to F2, suggesting a pattern primarily dominated by industrial input, while combustion sources were also non-negligible. This Zn input mainly originated from hot-dip galvanizing and zinc alloy die-casting in machinery manufacturing, as well as the electrochemical dissolution of Zn–Al–Cd sacrificial anodes [39,40]. PMF showed a significant covariance between Zn and Cd in F1 (Zn 58.85%, Cd 39.26%), consistent with the typical chemical composition of sacrificial anodes [41,42]. Among the four elements, Cu had the most complex sources. PMF analysis showed that its contribution to F1 was 57.14% and to F2 was 35.01%, with a ratio of approximately 1.6:1 between the two. Industrial sources might primarily include motor windings and electroplating wastewater, as well as marine corrosion inputs from Cu–Ni alloy piping and antifouling paints [43,44]. Combustion sources might mainly derive from coastal coal combustion and wear of motor vehicle components [45,46,47]. In addition, the notably high combustion contribution of Cu (35%) might be related to the dual industrial structure of the Zhejiang island sea area, characterized by electrical manufacturing plus energy consumption. Cd was the most diffusely distributed element. PMF results showed its contributions to F1 at 39.26%, F2 at 47.09%, and F3 at 13.65%, making it the only one among the nine elements with significant contributions across all three factors, with the combustion contribution slightly exceeding the industrial source. Industrial sources of Cd included the use of Cd electroplating coatings and the synergistic dissolution of Zn–Al–Cd sacrificial anodes [41,42,48], while combustion source might be related to coastal large-scale coal-fired power plant clusters and emissions from crude oil catalytic cracking at the Zhoushan Green Petrochemical Base [37,49].

PC2 accounted for 16.70% of the variance, with high loadings of V (0.90), Cr (0.86), and Ni (0.80) (Table S6, Figure 6). Compared with the background values of coastal sediments in Zhejiang (Table 1), only the average concentration of Ni was higher; the average concentration of V was consistent with the background value; while the Cr average concentration was lower. In addition, according to the *I*_geo_ values of metals (Table S4), Ni showed a certain degree of enrichment (average EF = 1.67), while Cr did not show obvious enrichment (average EF = 0.90). To test whether this factor grouping is controlled by lithogenic processes, we performed linear regression analysis of V, Cr and Ni against Ti as a conservative lithogenic element (Figure S2). The regression yielded R^2^ values of 0.45, 0.46, and 0.24, respectively (all *p* < 0.05), indicating that lithogenic processes might contribute to their enrichment but were not the dominant controlling factor. In PMF, Factor 2 (F2) was characterized by dominant contributions from V (63.66%), Ni (60.92%), and Cr (60.09%) (Figure 7). V and Ni are common metal porphyrin complexes in petroleum and are often used as indicators of ship emission sources [50,51]. The V/Ni ratio from heavy oil combustion typically exceeds 2.5, whereas that from gasoline vehicle emissions is below 2.5 [50,52]. In this study, the average V/Ni ratio in surface sediments was 3.18 ± 0.38 (Table S2). However, because V and Ni may also be released from petrochemical activities and other combustion-related processes, the V/Ni ratio should be regarded as supporting rather than definitive evidence of shipping-related emissions. The combustion–source association of V–Ni might be related to two important regional characteristics. First, the intensive shipping activities in the Ningbo–Zhoushan Port, where large ocean-going vessels burn heavy oil during berthing and port entry/exit, may release substantial amounts of V and Ni [50,51]. Second, during catalytic cracking and petroleum coke combustion at the Zhoushan Green Petrochemical Base, emissions of V and Ni may also occur [53,54]. The 60.09% contribution of Cr to F2 might be related to coal combustion in coastal power plants and petrochemical metallurgy [55]. Considering the correlations between V–Ni–Cr and Ti (Figure S2), F2 was likely a combustion-dominated source with a superimposed lithogenic background, rather than a purely anthropogenic or purely natural source. It should be noted that, the relative contributions of shipping emissions, petrochemical activities, and nature lithogenic processes cannot be quantitatively distinguished based solely on the present dataset.

PC3 accounted for 13.53% of the variance, with high loadings of Sr (0.87) and Ba (0.76) (Table S6, Figure 6), constituting a natural weathering input source. Compared with the background values of coastal sediments in Zhejiang, the average Sr concentration in sediments was slightly higher, while the average Ba concentration was slightly lower (Table 1). According to Tables S3 and S4, Sr showed a certain degree of enrichment but had not yet reached the pollution level, whereas Ba exhibited neither enrichment nor anthropogenic pollution. This source might reflect the natural input of alkaline earth metals from the weathering of Mesozoic volcanic rocks in the Zhejiang region to the sediments [56,57,58]. In PMF, Factor 3 (F3) was characterized by dominant contributions from Ba (49.69%) and Sr (40.12%) and was therefore identified as a natural weathering source (Figure 7). However, PMF revealed a noteworthy anomaly: Sr contributed as much as 39.90% to F2 (combustion source), nearly equal to its natural source contribution to F3 (40.12%). Previous studies have shown that during coal combustion, Sr tends to enrich in fly ash, which then undergoes long-range atmospheric transport and enters island sea area sediments through dry and wet deposition [59]. Additionally, Ba contributed 49.69% to F3 and 31.51% to F2; the latter might be related to petroleum drilling activities at the Zhoushan Green Petrochemical Base. Barite powder (BaSO_4_), used as a weighting agent in drilling fluids, is extensively consumed in oil and gas development, and losses during refining processes may lead to Ba release [60], thereby contributing to the natural background of Ba in the sediments.

## 4. Environmental Implications

The Zhejiang island sea area represents one of the most rapidly developing coastal regions in eastern China, characterized by intensive shipping activities, expanding port infrastructure, shipbuilding and repair industries, and growing petrochemical operations. Against this background, reliable baseline information on metal distributions are essential for distinguishing natural geochemical variability from anthropogenic influences and for evaluating long-term environmental changes associated with coastal development.

The combined PCA–PMF analysis identified three major sources of metals in surface sediments, namely industrial sources (29.92%), combustion-related sources (36.51%), and natural weathering sources (33.57%) (Figure 5). The predominance of anthropogenic contributions indicates that human activities have become an important factor influencing the regional geochemical environment. In particular, Pb–Zn–Cu–Cd was closely associated with industrial activities, whereas V–Ni–Cr was linked to combustion-related processes. These elemental assemblages may therefore serve as useful indicators for future environmental monitoring and source-tracking studies in the Zhejiang island sea areas. It is worth noting that, although this study employed PCA–PMF to quantitate source apportionment of metals in surface sediments, source apportionment itself is inherently complex. The reliability of the results depends, to a large extent, on the accurate determination of the mobile phases of metals derived from anthropogenic sources, and therefore further in-depth investigation is still needed.

An important contribution of this study is the establishment of a historical geochemical baseline based on sediment samples collected before the rapid expansion of large-scale petrochemical facilities, port construction, and intensified maritime activities that occurred during the last two decades. Such baseline information provides a valuable reference for future assessments of environmental change and enables comparisons with contemporary monitoring data to evaluate the long-term impacts of regional industrialization and coastal development.

Nevertheless, several uncertainties should be considered when interpreting the results. The present study primarily reflects historical environmental conditions rather than the current contamination status of the region. In addition, sediment properties that may influence metal accumulation, such as grain size distribution, organic matter content, and Fe–Mn oxide phases [61], were not available for evaluation. When assessing the risk of metal contamination in sediments, it is necessary not only to measure the total metal concentrations but also to determine the contents of their mobile phases, as the latter are the key factor controlling the mobility of metals at the sediment–water interface and their potential for secondary release [62]. Furthermore, although PCA–PMF analysis provides valuable information on potential source categories, the relative contributions of specific industrial sectors cannot be unequivocally distinguished without complementary evidence from emission inventories, atmospheric deposition measurements, isotopic tracers, or long-term monitoring datasets.

Future investigations should integrate contemporary sediment surveys with geochemical tracers, isotopic approaches, and environmental monitoring data to better quantify source contributions and evaluate temporal changes in metal contamination. Such efforts will improve our understanding of the environmental consequences of rapid coastal industrialization and support sustainable management of marine ecosystems in the Zhejiang island sea area.

## 5. Conclusions

This study investigated the concentrations, spatial distribution, pollution levels, and main sources of metals in surface sediments collected in 2008 from the Zhejiang island sea areas. The results showed that the metals exhibit spatial variability, with relatively similar distribution patterns observed for Cd, Cr, Cu, Ni, Pb and Zn. In addition, Ni, Sr, Cu, Zn and Cd showed a certain degree of enrichment, while Ni and Cd appeared to display characteristics of slight anthropogenic influence. Cd posed the highest ecological risk, although the overall ecological risk posed by the metal appeared to be low to moderate based on the concentration calculations. Source apportionment using PCA and PMF indicated that Cu, Zn, Pb, and Cd were primarily associated with inputs from coastal machinery manufacturing and shipbuilding; V, Ni, and Cr might be related to ship and fuel combustion emissions, with possible superimposed lithogenic contributions; and Sr and Ba appear to be predominantly derived from natural weathering. Meanwhile, the estimated contributions of the three sources accounted for 29.92%, 36.51%, and 33.57%, respectively. It should be noted that the source apportionment results were based on statistical receptor models and should be interpreted as indicative rather than definitive. Based on the metal concentrations of surface sediments collected in 2008 from the Zhejiang island sea areas, this study provides a historical baselines for metal distribution and ecological risks of metals in these sea areas, and offers a reference framework for future assessments. Given the lack of grain-size normalization in the risk calculations, further investigation using normalized approaches is recommended to confirm the observed patterns and source apportionment.

## Figures and Tables

**Table 2:** Comparison of metal concentrations in surface sediments between Zhejiang islands and other sea areas (mg/kg).

Location	Ni	Cr	Cu	Zn	Cd	Pb	Reference
Study area	35.70	88.81	29.01	97.08	0.23	26.30	This study
South coast of Zhejiang, China	45.07	55.46	28.15	115.87	0.13	29.40	[12]
Lianyungang offshore, China	21.20	41.40	-	49.60	0.53	12.40	[4]
Hebei coastal area, China	-	48.80	16.10	50.00	0.10	19.40	[26]
Shandong Peninsula coastal area, China	-	71.47	21.19	80.77	0.19	24.37	[15]
Fujian Coastal area, China	27.40	57.40	-	95.90	0.08	37.10	[27]
Yellow River estuary	-	65.40	32.40	81.10	0.27	31.90	[28]
Yellow Sea (2013)	-	48.64	21.17	70.92	0.28	16.71	[28]
Bohai Sea (2013)	-	54.67	28.80	88.40	0.36	19.67	[28]
Marine Quality Standard Grade I	-	80.00	35.00	150.00	0.50	60.00	[29]
Marine Quality Standard Grade II	-	150.00	100.00	350.00	1.50	130.00	[29]
≤Marine Quality Standard Grade I	-	26.83%	90.24%	97.56%	98.78%	97.56%	This study

## Data Availability

The original contributions presented in this study are included in the article/Supplementary Material. Further inquiries can be directed to the corresponding author(s).

## Supplementary Materials

The following supporting information can be downloaded at https://www.mdpi.com/article/10.3390/toxics14080669/s1. Table S1: The proportions of metals in three factors; Table S2: Metal concentrations; Table S3: EF values; Table S4: *I*_geo_ values; Table S5: $E_{r}^{i}$ and *RI* values of metals; Table S6: Principal component analysis (PCA) of metals in surface sediments from the Zhejiang island sea areas; Figure S1: Relationship between Ti and *I*_geo_ (Cd) and *I*_geo_ (Ni); Figure S2: Relationship between Ti and Ni, Cr, and V.
