# Supplementary material for: Anthropogenic Versus Natural Controls on Metals in Surface Sediments from the Zhejiang Island Sea Area: Evidence from Historical Baseline and PMF Analysis
Source: Toxics. 2026 Jul 28;14(8):669. doi: 10.3390/toxics14080669 (PMC13517411; doi:10.3390/toxics14080669)
Supplement: Supplementary file 1 [file toxics-14-00669-s001.zip › toxics-4422459-supplementary.pdf]

Anthropogenic Versus Natural Controls on Metals in Surface Sediments from the Zhejiang Island Sea Area: Evidence from Historical Baseline and PMF Analysis

Ruirui Wang [1], Yanlong Gan [1], Jian Hu [1], Xinyu Mi [1], Jianyu Ni [2], Zhiyong Liu [3], Wenhan Cheng [4,*] and Chuanyu Chang [5,*]

1 School of the Environment and Safety Engineering, Jiangsu University, Zhenjiang 212013, China

2 Key Laboratory of Submarine Geosciences, Second Institute of Oceanography, Ministry of Natural Resources, Hangzhou 310012, China

3 State Key Laboratory of Radiation Medicine and Protection, School of Radiation Medicine and Protection, Collaborative Innovation Center of Radiological Medicine of Jiangsu Higher Education Institutions, Biomedical Basic Research Center (BBRC) of Jiangsu, Soochow University, Suzhou 215123, China

4 Anhui Provincial Key Laboratory of Hazardous Factors and Risk Control of Agri-Food Quality Safety, College of Resource & Environment, Anhui Agricultural University, Hefei 230036, China

5 Laboratory of Karst Environmental Evolution and Ecological Security, Institute of Geochemistry, Chinese Academy of Sciences, Guiyang 550081, China

* Correspondence: chengwh@ahau.edu.cn (W.C.); changchuanyu@mail.gyig.ac.cn (C.C.)

Table S1. The proportions of metals in three factors (%) in surface sediments from the Zhejiang island sea areas.

	F1	F2	F3
Sr	18.46	18.36	9.20
Ni	0.94	6.54	3.25
V	5.04	21.23	7.08
Cr	2.95	16.18	7.79
Cu	0.72	3.20	5.23
Zn	3.84	8.94	18.28
Cd	0.01	0.03	0.03
Ba	66.61	25.21	42.24
Pb	1.43	0.29	6.90

Table S2. Metal concentrations in surface sediments from the Zhejiang island sea areas.

Island	Location	Sample	Property	Sr mg/kg	Ni mg/kg	V mg/kg	Cr mg/kg	Cu mg/kg	Zn mg/kg	Cd mg/kg	Ba mg/kg	Pb mg/kg	Ti mg/kg
Xiushan	Zhoushan City	1	Mud, Silt	149.80	38.78	122.85	91.12	32.68	100.15	0.25	415.17	32.43	5401.45
		2	Mud	214.82	37.17	116.83	89.43	30.01	95.08	0.25	388.89	22.76	5236.12
		3	Mud	164.86	42.40	132.35	100.83	34.59	117.48	0.27	450.98	27.39	5684.89
Zhoushan	Zhoushan City	4	Mud	150.56	46.63	141.02	108.84	41.60	137.11	0.30	480.04	45.27	5630.28
		5	Mud	148.50	46.32	139.50	105.43	39.78	118.51	0.26	442.35	30.15	5583.07
		6	Mud	162.48	38.33	119.46	91.10	32.75	108.44	0.28	428.86	24.78	5367.03
		7	Mud	158.27	43.37	133.44	102.14	36.13	114.78	0.26	445.00	27.83	5754.33
		8	Mud	159.02	41.07	126.26	97.43	33.13	108.08	0.23	437.22	25.32	5627.83
		9	Mud	156.21	45.56	118.74	114.82	33.82	104.88	0.29	418.54	24.05	5575.60
		10	Mud	157.07	40.35	117.59	99.82	32.67	101.29	0.30	443.53	25.82	5559.00
		11	Mud	155.91	41.86	129.48	99.09	38.21	114.00	0.28	430.45	26.98	5548.84
		12	Mud	156.98	43.52	126.40	107.18	35.91	111.23	0.28	428.32	25.97	5544.36
		13	Mud	155.09	42.96	126.21	106.51	34.41	108.23	0.29	432.79	25.83	5959.59
		14	Mud	139.56	32.67	96.87	80.83	26.54	81.61	0.21	435.02	21.26	4628.31
		15	Mud	131.46	30.42	87.66	76.21	23.33	79.72	0.22	438.68	23.52	4266.49
		16	Mud	159.92	39.25	115.01	98.86	31.41	112.14	0.26	422.63	23.04	5651.51
		17	Mud	152.00	34.67	100.04	87.45	24.76	81.66	0.22	386.04	20.72	5113.45
		18	Mud	153.83	31.66	108.52	82.80	24.63	83.84	0.21	423.52	22.47	5327.47
		19	Mud	131.49	30.57	102.48	80.23	24.33	82.47	0.21	379.06	21.55	5377.11
		20	Sandy mud	163.40	41.79	107.99	109.34	24.34	87.30	0.22	424.21	19.33	5641.52
		21	Mud, Silt	166.67	37.36	107.42	93.73	26.29	90.86	0.23	406.58	20.64	5512.92

		22	Mud	129.56	40.11	131.84	96.65	31.98	113.63	0.22	421.48	29.60	5100.57
		23	Mud	123.35	36.65	122.98	90.95	29.12	99.04	0.20	388.06	26.68	4797.15
		24	Mud	118.60	37.91	125.26	91.38	30.86	108.17	0.22	392.36	31.09	4722.58
Cezi	Zhoushan City	25	Silty mud	136.53	27.21	91.23	71.65	19.38	69.03	0.19	364.04	18.58	4555.51
Daxie	Ningbo City	26	Silt	154.18	70.26	94.19	139.47	82.48	242.40	0.37	643.84	111.89	4695.95
Jintang	Zhoushan City	27	Sandy mud	135.48	29.84	101.34	79.89	23.16	91.31	0.23	439.86	23.63	4854.06
		28	Sandy mud	141.53	26.22	90.86	70.45	18.50	68.06	0.20	368.08	17.47	4795.20
		29	Sandy mud	136.49	27.00	90.99	70.78	19.44	70.58	0.20	362.17	17.87	4578.70
Aoshan	Zhoushan City	30	Silty mud	162.91	28.56	102.72	82.73	22.53	80.85	0.21	403.10	21.64	5831.88
		31	Silty mud	167.68	25.46	91.35	76.65	17.46	66.26	0.19	389.42	17.80	5397.59
		32	Silty mud	162.08	26.90	94.40	77.78	18.69	68.75	0.22	399.33	18.21	5386.56
Putuo	Zhoushan City	33	Mud	133.17	37.31	124.87	94.60	33.05	116.63	0.25	438.14	36.86	5455.67
		34	Mud	146.96	35.30	119.20	91.46	29.56	94.61	0.21	408.63	24.92	5553.54
		35	Mud	151.56	32.00	107.03	82.72	25.72	86.37	0.18	399.90	22.18	5314.18
		36	Sandy mud	121.31	18.47	72.44	55.16	15.28	57.98	0.10	404.91	18.93	3748.16
		37	Sandy mud	150.07	32.06	109.00	81.79	25.53	84.84	0.17	409.39	22.34	5166.12
		38	Sandy mud	154.19	30.95	105.78	82.90	24.17	81.97	0.19	386.70	20.56	5354.70
Dengbu	Zhoushan City	39	Sandy mud	165.06	31.50	107.41	88.36	25.61	85.95	0.19	412.80	21.75	5637.54
		40	Sandy mud	164.65	31.54	107.23	84.05	24.38	83.92	0.19	405.47	20.75	5574.25
Zhujiajian	Zhoushan City	41	Mud	136.48	17.96	75.00	59.27	28.82	123.10	0.29	350.62	40.18	4514.56
		42	Mud	128.57	24.47	87.47	63.23	22.48	75.42	0.16	383.26	22.03	4903.63
		43	Mud	161.94	31.65	108.90	89.63	25.51	84.57	0.22	402.94	20.88	5991.15
Daorenshan	Ningbo City	44	Mud	151.77	34.79	109.81	82.24	28.69	95.08	0.22	401.16	22.01	5251.57
		45	Mud	161.72	33.91	105.60	83.41	26.61	88.91	0.22	404.35	23.39	5367.50

		46	Silt to mud	164.06	29.08	78.42	61.10	19.98	72.26	0.16	566.67	23.09	4456.54
Meishan	Ningbo City	47	Mud	152.54	33.76	104.92	79.82	27.87	91.88	0.21	435.30	22.00	4996.72
		48	Mud	140.37	32.58	100.38	78.43	25.75	84.31	0.19	364.17	22.88	4805.82
		49	Mud	146.07	33.58	104.85	79.82	27.46	90.87	0.19	383.59	23.43	4947.43
		50	Mud	131.23	41.84	126.08	94.56	36.08	113.28	0.24	394.91	27.31	4974.49
		51	Mud	128.24	40.95	122.27	91.50	34.98	105.60	0.22	378.80	26.04	4795.03
		52	Mud	123.74	45.36	126.98	95.20	38.07	110.98	0.25	380.50	28.11	4882.23
Fodu	Zhoushan City	53	Sandy mud	158.65	22.86	73.66	53.56	28.55	79.56	0.23	441.30	21.30	3876.77
		54	Mud	136.58	32.09	97.46	73.98	25.44	87.50	0.19	375.86	22.93	4505.54
		55	Mud	141.65	31.22	99.04	75.88	24.73	82.00	0.20	367.48	21.36	4856.11
Liuheng	Zhoushan City	56	Sandy mud	151.05	29.69	96.07	81.29	24.29	94.53	0.18	516.51	48.82	4986.31
		57	Sandy mud	149.65	39.12	119.67	99.96	31.24	195.12	0.67	430.68	66.21	5747.75
		58	Mud	161.30	42.61	123.21	106.72	31.12	101.06	0.19	431.44	25.79	6007.35
		59	Mud, Silt	165.38	43.11	121.87	108.62	30.99	100.20	0.26	441.98	24.58	5648.02
		60	Mud, Silt	181.27	45.56	121.18	121.14	29.02	99.68	0.26	469.61	22.85	6199.20
		61	Sandy mud	141.22	38.76	106.45	101.53	26.46	92.02	0.25	361.49	21.21	5585.43
		62	Silty mud	198.02	42.65	114.79	104.46	32.22	99.60	0.23	415.84	26.81	4891.50
		63	Silty mud	150.20	46.69	131.19	114.29	34.63	108.51	0.25	426.71	27.35	5750.63
		64	Silty mud	154.06	42.95	127.61	103.88	33.45	103.35	0.25	420.98	25.82	5661.49
		65	Silty mud	267.45	38.35	107.91	90.84	28.58	85.54	0.23	349.20	21.49	5328.21
		66	Silty mud	195.95	40.20	130.60	111.02	30.21	99.15	0.26	458.11	24.03	7547.15
Taohua	Zhoushan City	67	Silty mud	220.74	16.10	70.89	44.31	14.29	65.11	0.14	1076.45	21.14	4125.88
		68	Silty mud	150.85	32.72	109.87	85.65	27.20	87.62	0.18	437.30	23.19	5093.25

		69	Mud	161.98	34.94	118.00	88.85	26.85	88.89	0.20	416.07	21.32	5576.16
Gaotang	Ningbo City	70	Mud	151.54	38.91	128.08	97.11	34.81	107.16	0.19	476.83	31.08	5688.57
		71	Mud	150.78	38.27	125.39	92.15	32.42	101.04	0.18	409.74	26.44	5478.04
Huao	Ningbo City	72	Mud	134.84	29.34	99.55	74.88	22.05	78.32	0.19	363.44	21.15	4619.17
		73	Mud	150.91	32.06	110.50	81.81	24.46	81.93	0.23	392.06	22.68	5031.81
		74	Mud	130.21	34.08	115.78	86.25	27.33	88.76	0.21	385.49	24.89	4989.20
Nantian	Ningbo City	75	Mud	124.00	28.42	99.45	74.50	22.01	78.65	0.20	362.86	22.04	4612.72
		76	Mud	123.35	26.79	99.82	76.27	20.57	79.05	0.18	372.88	20.06	4667.31
		77	Mud	136.29	36.20	119.59	89.82	31.72	101.59	0.17	398.50	26.13	4976.83
		78	Mud	141.09	36.74	121.66	91.74	31.58	99.53	0.17	407.56	26.29	5122.88
		79	Mud	144.37	39.16	127.68	94.57	33.44	110.52	0.19	423.60	27.72	5292.55
Tantoushan	Ningbo City	80	Sandy mud	148.23	34.58	112.51	79.11	31.43	103.72	0.21	657.37	29.17	5032.58
		81	Sandy mud	146.19	41.47	126.38	95.87	34.10	108.33	0.24	416.82	26.11	5438.98
		82	Sandy mud	151.59	39.89	124.11	91.41	33.29	106.61	0.23	410.91	25.64	5405.01

Table S3. EF values of metals in surface sediments from the Zhejiang island sea areas.

EF	Sr	Ni	V	Cr	Mn	Cu	Zn	Cd	Ba	Pb
Max	2.18	3.66	1.20	1.57	1.39	3.02	2.73	5.19	2.68	4.41
Min	0.99	0.95	0.75	0.57	0.53	0.56	0.65	1.15	0.62	0.59
Average	1.20	1.67	0.96	0.90	0.90	0.96	0.99	1.93	0.85	0.94
Percentage of stations with EF>1	98.78%	97.56%	35.37%	19.51%	23.17%	39.02%	40.24%	100%	9.76%	23.17%

Table S4. I_{geo} values of metals in surface sediments from the Zhejiang island sea areas.

I_{geo}	Sr	Ni	V	Cr	Cu	Zn	Cd	Ba	Pb
Max	0.57	1.23	–0.20	0.01	0.95	0.81	2.02	0.59	1.50
Min	–0.60	–0.90	–1.19	–1.64	–1.58	–1.26	–0.77	–1.03	–1.18
Average	–0.25	0.22	–0.56	-0.66	–0.60	–0.55	0.42	–0.76	–0.66
Percentage of stations with I_{geo} > 0	7.32%	79.27%	0	1.22%	1.22%	2.44%	95.12%	1.22%	6.10%

Table S5. E_r^i and *RI* values of metals in surface sediments from the Zhejiang island sea areas.

	E_r^i						*RI*
	Ni	Cr	Cu	Zn	Cd	Pb	
Max	17.57	3.02	14.52	2.62	183.12	21.19	215.21
Min	4.02	0.96	2.52	0.63	26.47	3.31	39.19
Average	8.92	1.92	5.11	1.05	61.77	4.98	83.76
$E_r^i < 40$	100%	100%	100%	100%	2.44%	100%	-
$40 < E_r^i < 80$	-	-	-	-	51.22%	-	-
$80 < E_r^i < 160$	-	-	-	-	3.66%	-	-
$160 < E_r^i < 320$	-	-	-	-	1.22%	-	-

Table S6. Principal component analysis (PCA) of metals in surface sediments from the Zhejiang island sea areas.

Components	Initial Eigenvalues			Metals	Component matrix		
	Total	Variance%	Cumulative %		PC1	PC2	PC3
1	4.86	53.95	53.95	Pb	0.97	0.03	0.06
2	1.50	16.70	70.64	Zn	0.92	0.35	0.01
3	1.22	13.53	84.17	Cu	0.78	0.50	0.05
4	0.70	7.79	91.97	Cd	0.68	0.29	0.00
5	0.42	4.65	96.62	V	0.11	0.90	–0.09
6	0.17	1.86	98.48	Cr	0.41	0.86	0.06
7	0.09	1.04	99.52	Ni	0.54	0.80	0.07
8	0.03	0.30	99.81	Sr	–0.16	0.22	0.87

9	0.02	0.19	100.00	Ba	0.30	–0.30	0.76
				Eigenvalues	4.86	1.50	1.22
				% total variance	53.95	16.70	13.53
				%cumulative	53.95	70.64	84.17

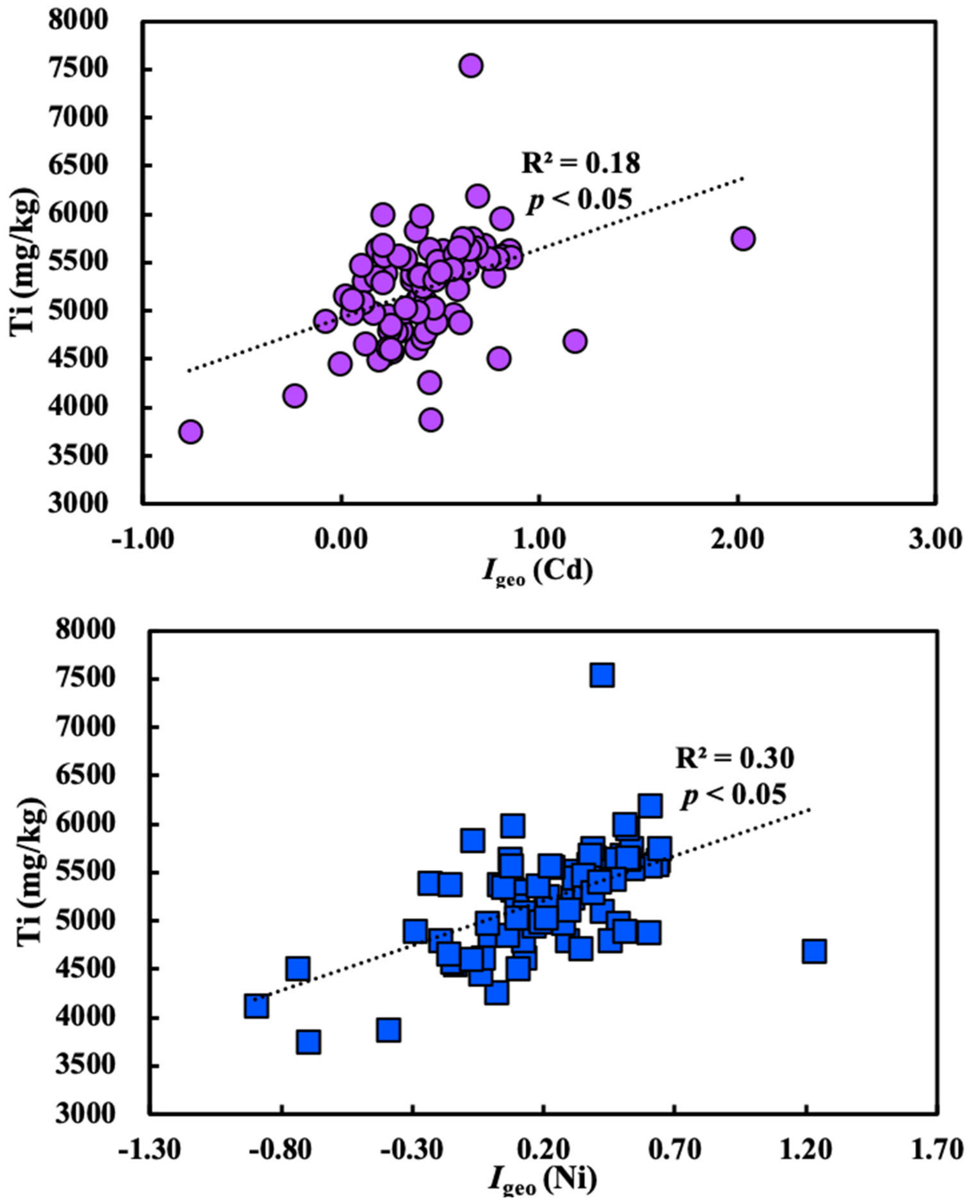

Figure S1. The relationship between Ti and I_{geo} (Cd), I_{geo} (Ni).

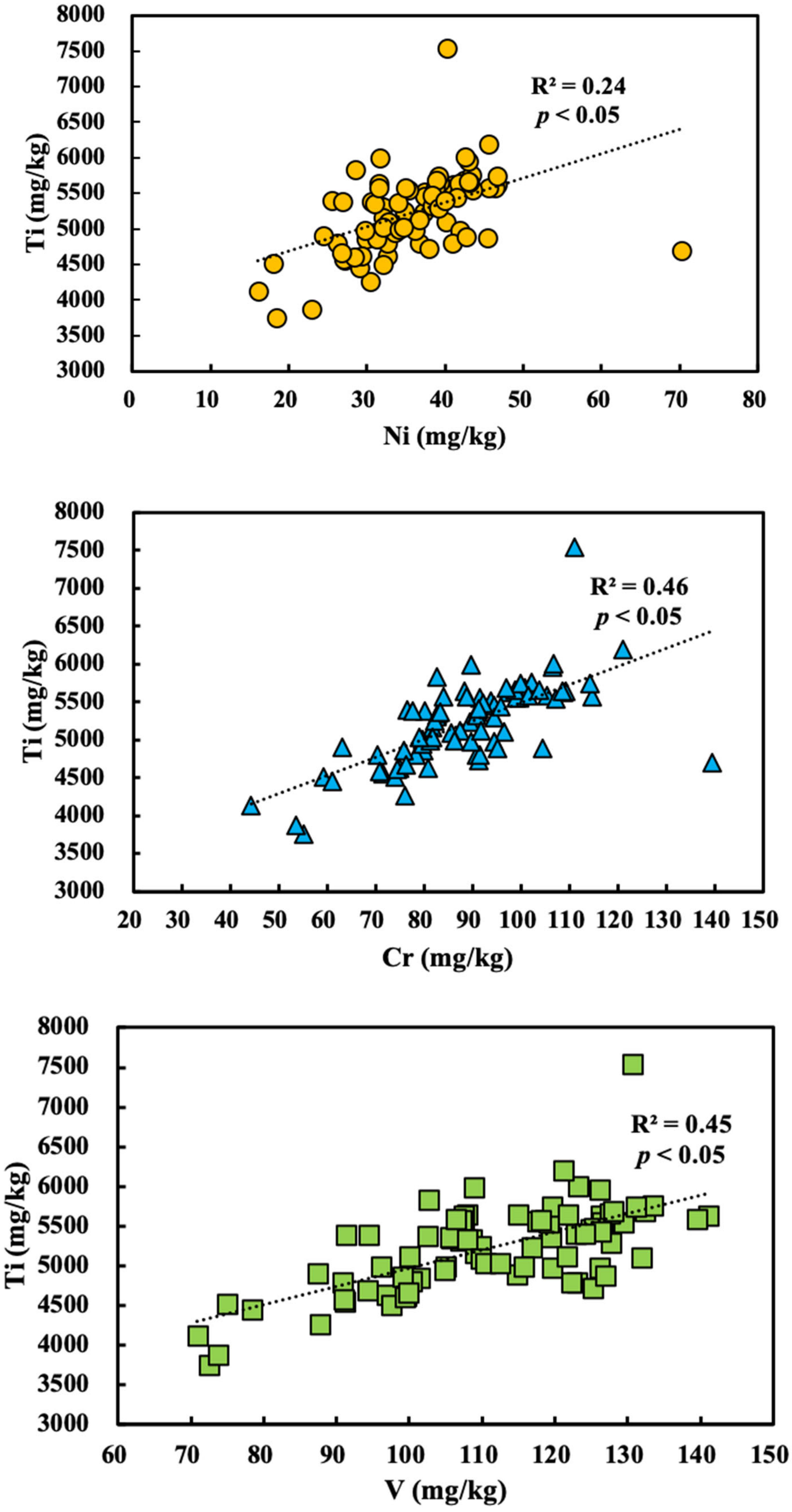

Figure S2. The relationship between Ti and Ni, Cr, V.
